# Anti-correlation of KLRG1 and PD-1 expression in human tumor CD8 T cells

**DOI:** 10.18632/oncotarget.28679

**Published:** 2025-01-20

**Authors:** Steven A. Greenberg

**Affiliations:** ^1^Department of Neurology, Brigham and Women’s Hospital, Harvard Medical School, Boston, MA 02115, USA

**Keywords:** immunotherapy, KLRG1

## Abstract

Recently, combination checkpoint therapy of cancer has been recognized as producing additive as opposed to synergistic benefit due in part to positively correlated effects. The potential for uncorrelated or negatively correlated therapies to produce true synergistic benefits has been noted. Whereas the inhibitory receptors PD-1, CTLA-4, TIM-3, LAG-3, and TIGIT have been collectively characterized as exhaustion receptors, another inhibitory receptor KLRG1 was historically characterized as a senescent receptor and received relatively little attention as a potential checkpoint inhibitor target. The anti-tumor effects of KLRG1 blockade has relatively recently been demonstrated in preclinical *in vivo* studies. Here, expression of the inhibitory receptors PD-1, CTLA-4, TIM-3, LAG-3, TIGIT, and KLRG1 was studied in publicly available gene expression datasets. Bulk RNA microarray and RNAseq, and single cell RNAseq data from healthy blood and tumor tissue samples were analyzed for Pearson correlation. CD8 T cell differentiation of memory T cells from the TEM to TEMRA states is characterized by PD-1/KLRG1 anti-correlation, with decreased PD-1 expression but increased KLRG1 expression. Single cell RNAseq analysis of tumor infiltrating CD8 T cells shows positive correlation of CTLA-4, TIM-3, LAG-3, TIGIT, GITR, 4-1BB, and OX40 with PD-1 but negative correlation of KLRG1 with PD-1. The anti-correlation of PD-1 and KLRG1 expression in human tumor infiltrating CD8 T cells suggests the potential for combination therapy supra-additive benefits of anti-PD-1 and anti-KLRG1 therapies.

## INTRODUCTION

Targeting of the T cell coinhibitory receptors PD-1, CTLA-4, TIM-3, LAG-3, and TIGIT and costimulatory receptors GITR, 4-1BB, and OX40 for applications in oncology has gained widespread interest [1–3] and been tested in clinical studies. Therapeutic benefits in preclinical mouse studies with blocking antibodies was demonstrated for CTLA-4 by 1996 [4], PD-1 by 2005 [5, 6], LAG-3 by 2007 [7], TIM-3 by 2013 [8], and TIGIT by 2014 [9].

In contrast, the inhibitory receptor KLRG1 has received less interest. A benefit for KLRG1 blockade in mouse models was demonstrated in 2019 and again in 2021 [10, 11], with KLRG1 and PD-1 blockade producing supra-additive benefits in the B16F10 mouse melanoma model.

Historically, the pursuit of additional T cell inhibitory receptors has been driven by attention to co-regulated expression. Thus, studies in 2007 of the molecular signature of mouse CD8+ T cells during chronic viral infection identified coordinate regulation of all inhibitory receptors studied (including PD-1, CTLA-4, and LAG-3) except for two, KLRG1 and KLRA9 [12]. Subsequent nearest neighbor correlation studies of this data identified KLRG1 as uniquely anti-correlated with PD-1 in mouse CD8+ T cells during chronic viral infection [13]. The coordinated expression of PD-1 with TIGIT [9] has also been emphasized.

Ultimately, a view emerged that receptors with coordinated expression to each other constituted a class of exhaustion receptors that were promising therapeutic targets while receptors with low or negative correlation to PD-1 might not be attractive targets. This view was further cemented by 2011 and 2015 with nomenclature for cancer CD8 T cells characterizing CTLA-4, PD-1, LAG-3, and TIM-3 as marking “exhausted” cells, whereas KLRG1 marked senescent cells [14, 15]. Alternative views as early as 2012 have noted the arbitrary distinction between exhaustion and senescence applied to these receptors, that human CD8 T cell inhibitory receptor surface protein expression may depend on differentiation and that KLRG1 protein surface expression is much higher in CD8 effector memory and effector populations than PD-1, CTLA-4, TIM-3, and LAG-3 [16, 17].

Here, we point out the distinct anti-correlation of KLRG1 expression with PD-1 and other checkpoint receptors in human CD8 T cell tumor infiltrating lymphocytes (TILS). Combination therapy in cancer seeks to increase efficacy over monotherapy, ideally through synergy that produces more than just additive benefits of two independent treatments. However, combination therapy synergistic efficacy has very uncommonly been seen in cancer immunotherapy combination trials [18]. Previously the potential for combination therapies of anti-correlated combinations to produce clinical benefits greater than the sum of the benefits of the individual therapies has been noted [19]. As this effect has been unrealized in the field of combination inhibitory receptor blocking therapies in the treatment of cancer, the identification of anti-correlated mechanisms may yield synergistic combination therapy benefits beyond those seen to date.

## RESULTS

### KLRG1 marks highly differentiated cytotoxic T cells that do not express PD-1 in healthy human blood and tumor

**Figure 1:**
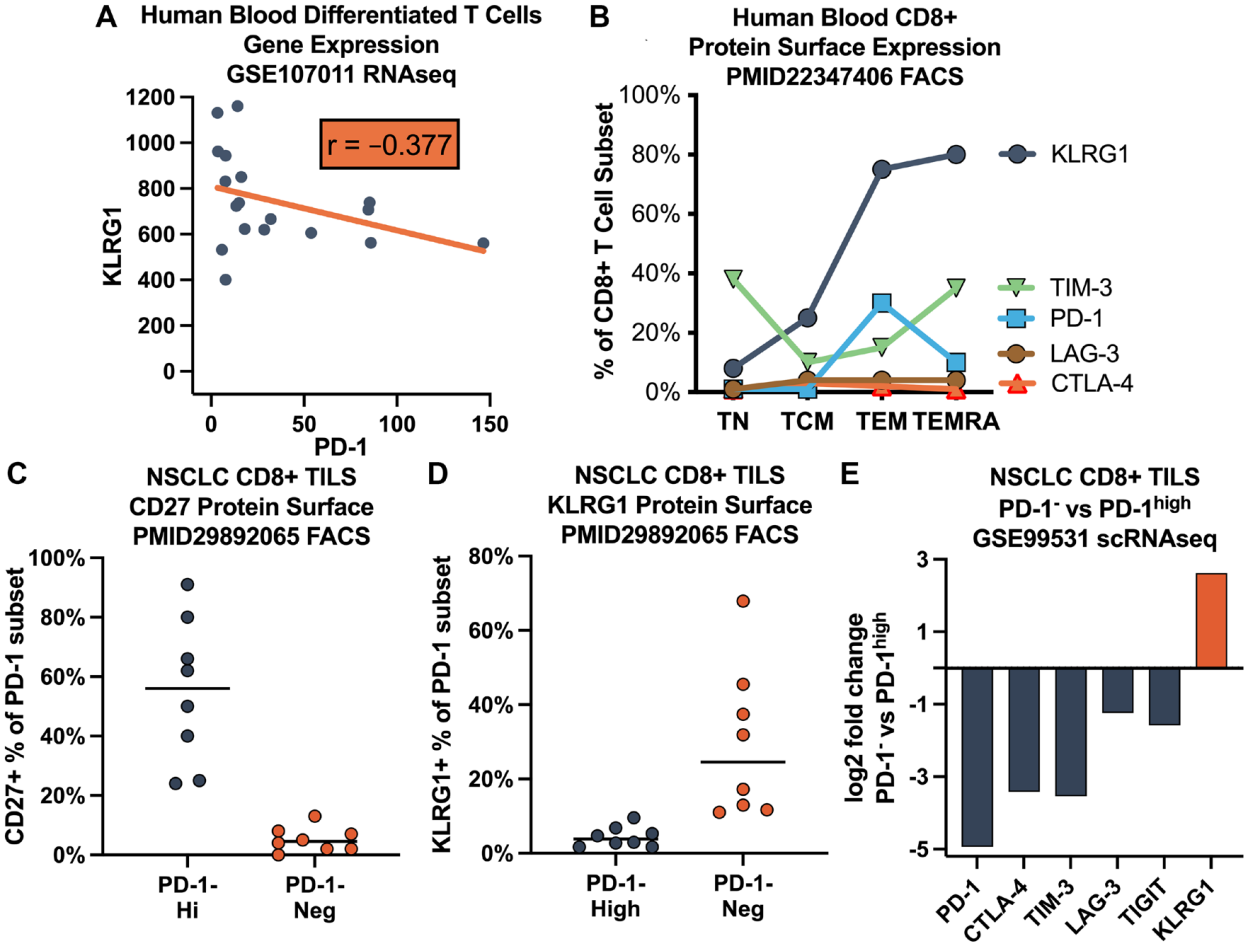
Distinct expression pattern of KLRG1 on highly differentiated human T cells. (**A**) Anticorrelated gene expression with PD-1 in RNAseq dataset GSE107011 for differentiated T cell subsets CD4 TEMRA, CD8 TEM, CD8 TEMRA, and γδ T cells. (**B**) Abstracted previously published data [17] for human blood CD8+ T cells protein surface expression showing increased KLRG1 on effector T memory (TEM) and effector (TEMRA) cells contrasting with decreasing PD-1 on TEMRA. (**C**–**E**) Characterization of PD-1+ CD8+ tumor infiltrating lymphocytes (TILS) in non-small cell lung cancer (NSCLC) datasets. (C) Abstracted data from previous publication [20] demonstrating that PD-1-high TILS are not highly differentiated TEMRA (CD27+) and (D) that KLRG1+ TILS are PD-1-. (E) Public domain single cell RNAseq (GSE99531) shows coregulated expression of CTLA-4, TIM-3, LAG-3, and TIGIT but anti-regulated KLRG1 expression in PD-1-high TILS.

Across the highly differentiated T cell population in normal blood, KLRG1 gene expression was anti-correlated (negatively correlated) with PD-1 (*r* = −0.377) (Figure 1A). Previous authors have noted that KLRG1 protein surface expression by flow cytometry increases substantially in TEM and TEMRA subsets (75–80% of CD8+ T cells), while PD-1 expression decreases from TEM (30%) to TEMRA (10%) subsets [17] (Figure 1B).

A similar pattern has been reported [20] in non-small cell lung cancer (NSCLC) CD8+ tumor infiltrating lymphocytes (TILS), where PD-1-high cells are earlier stage CD27+ T cells, while KLRG1+ cells are later-stage CD27– T cells that are not PD-1-high (Figure 1C, 1D). More generally, NSCLC CD8+ PD-1-high vs. PD-1-negative TILS have coregulated expression of CTLA-4, TIM-3, LAG-3, and TIGIT, but anti-regulated expression of KLRG1 (Figure 1E).

### Tumor infiltrating CD8 T cell expression of multiple coinhibitory and costimulatory checkpoint receptors are positively correlated to PD-1, while KLRG1 expression is anti-correlated to PD-1

**Figure 2:**
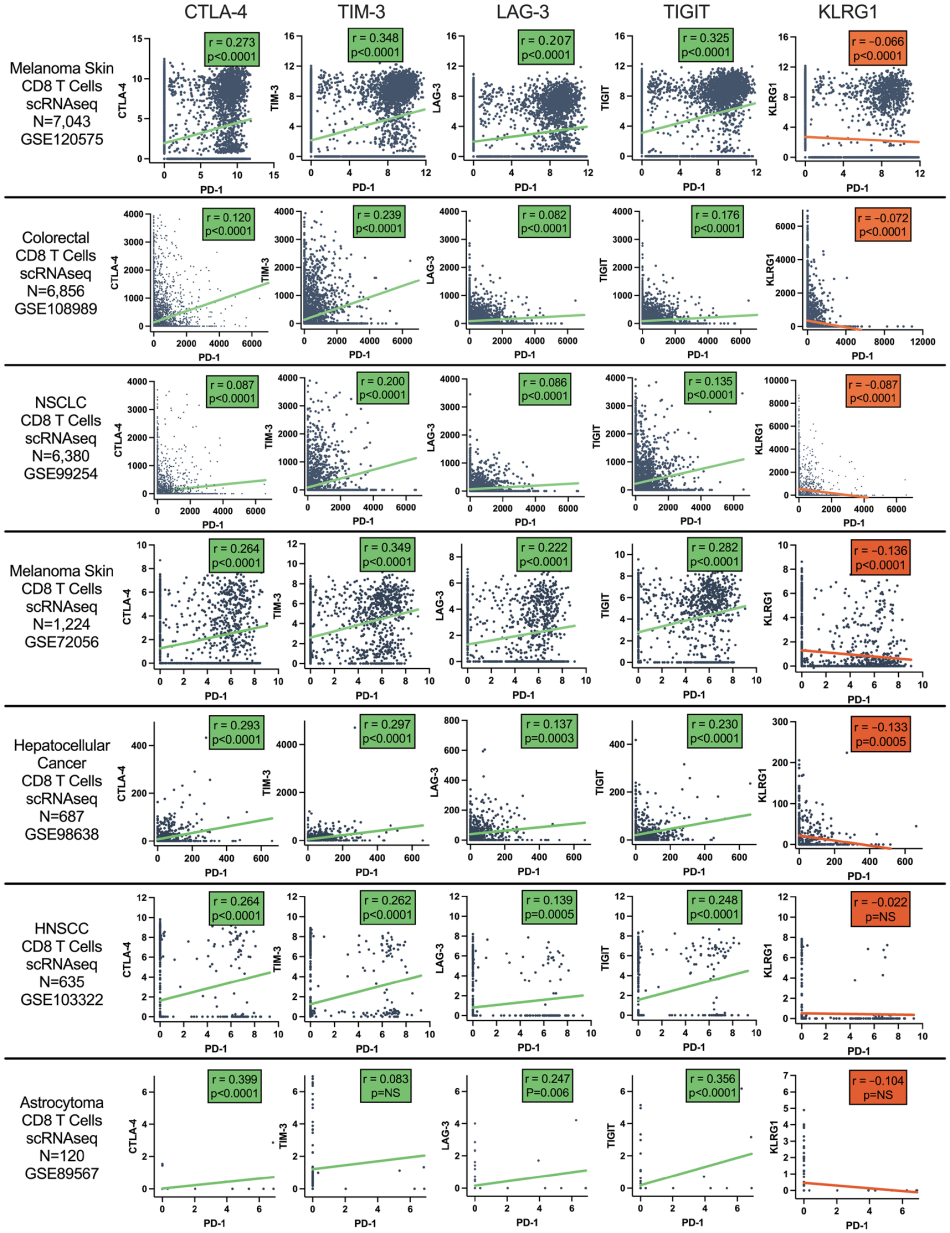
Pearson correlation of CD8 T cell co-inhibitory receptor gene expression with PD-1 across CD8 T cells from tumor samples. Data from single cell RNAseq datasets of melanoma, colorectal cancer, NSCLC, hepatocellular carcinoma (HCC), head and neck squamous cell cancer (HNSCC), and astrocytoma.

To look more broadly at coinhibitory receptor (CTLA-4, TIM-3, LAG-3, TIGIT, and KLRG1) and costimulatory receptor (GITR, OX40, and 4-1BB) CD8 tumor expression correlation with PD-1, public domain single cell RNAseq gene expression datasets with available processed gene counts were obtained, and CD8 T cell data analyzed. Datasets of melanoma (*N* = 7,043 CD8 T cells), colorectal cancer (*N* = 6,856 CD8 T cells), non-small cell lung cancer (*N* = 6,380 cells), melanoma (*N* = 1,224 CD8 T cells), hepatocellular carcinoma (*N* = 687 CD8 T cells), head and neck squamous cell carcinoma (*N* = 635 CD8 T cells), and astrocytoma (*N* = 120 CD8 T cells) were analyzed. This showed positive correlation of all coinhibitory, except KLRG1, and all costimulatory receptor expression with PD-1, while KLRG1 was uniquely anti-correlated with PD-1. (Figure 2 and Table 1).

**Table 1:** Pearson correlation coefficients *r* and *p*-values of CD8 T cell coinhibitory and costimulatory receptor expression with PD-1 across various single cell RNAseq datasets

Dataset		GSE120575 Melanoma	GSE108989 Colorectal	GSE99254 NSCLC	GSE72056 Melanoma	GSE98638 HCC	GSE103322 HNSCC	GSE89567 Astrocytoma
**# CD8 T Cells**		7,043	6,856	6,380	1,224	687	635	120
**PD-1 vs. CTLA-4**	***r***	0.273	0.120	0.080	0.264	0.293	0.211	0.399
	***p***	^****^	^****^	^****^	^****^	^****^	^****^	^****^
**PD-1 vs. TIM-3**	***r***	0.348	0.239	0.196	0.282	0.297	0.262	0.083
	***p***	^****^	^****^	^****^	^****^	^****^	^****^	NS
**PD-1 vs. LAG-3**	***r***	0.207	0.082	0.086	0.223	0.137	0.139	0.247
	***p***	^****^	^****^	^****^	^****^	^***^	^***^	^**^
**PD-1 vs. TIGIT**	***r***	0.325	0.176	0.135	0.349	0.230	0.248	0.356
	***p***	^****^	^****^	^****^	^****^	^****^	^****^	^****^
**PD-1 vs. GITR**	***r***	0.100	0.056	0.075	0.089	0.194	0.183	0.037
	***p***	^****^	^****^	^****^	^**^	^****^	^****^	NS
**PD-1 vs. 4-1BB**	***r***	0.258	0.088	0.088	0.281	0.218	0.152	0.676
	***p***	^****^	^****^	^****^	^****^	^****^	^***^	^****^
**PD-1 vs. OX40**	***r***	0.048	0.020	0.044	0.011	0.089	0.057	−0.030
	***p***	^****^	NS	^***^	NS	^*^	NS	NS
**PD-1 vs. KLRG1**	***r***	−0.066	−0.072	−0.087	−0.136	−0.133	−0.022	−0.104
	***p***	^****^	^****^	^****^	^****^	^***^	NS	NS

*p*-value notation ^****^ = <0.0001, ^***^ = <0.001, ^**^ = <0.01, ^*^ = <0.05.

**Figure 3:**
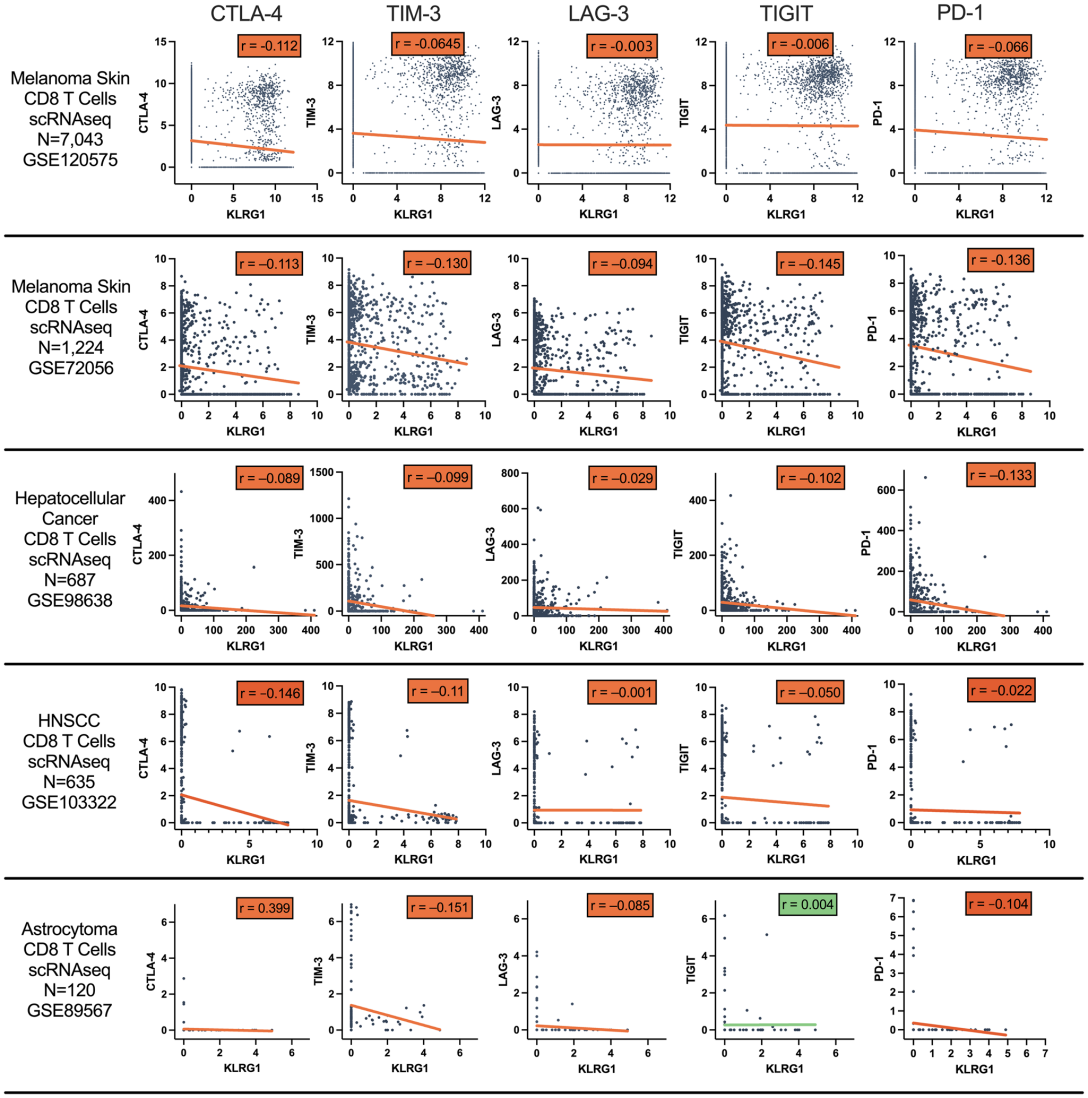
Pearson correlation of CD8 T cell co-inhibitory receptor gene expression with KLRG1 across CD8 T cells from tumor samples. Data from single cell RNAseq datasets of melanoma, colorectal cancer, NSCLC, hepatocellular carcinoma (HCC), head and neck squamous cell cancer (HNSCC), and astrocytoma.

KLRG1 expression in CD8+ TILS datasets were additionally broadly anti-correlated with coinhibitory receptors CTLA-4, TIM-3, LAG-3, and TIGIT and costimulatory receptors GITR, OX40, and 4-1BB (Figure 3 and Table 2).

**Table 2:** Pearson correlation coefficients *r* and *p*-values of CD8 T cell coinhibitory and costimulatory receptor expression with KLRG1 across various single cell RNAseq datasets

Dataset		GSE120575 Melanoma	GSE108989 Colorectal	GSE99254 NSCLC	GSE72056 Melanoma	GSE98638 HCC	GSE103322 HNSCC	GSE89567 Astrocytoma
**# CD8 T Cells**		7,043	6,856	6,380	1,224	687	635	120
**KLRG1 vs. PD-1**	***r***	−0.066	−0.072	−0.087	−0.136	−0.133	−0.022	−0.104
	***p***	^****^	^****^	^****^	^****^	^***^	NS	NS
**KLRG1 vs. CTLA-4**	***r***	−0.112	−0.120	−0.088	−0.113	−0.089	−0.146	−0.071
	***p***	^****^	^****^	^****^	^****^	^*^	^***^	NS
**KLRG1 vs. TIM-3**	***r***	−0.065	−0.078	−0.099	−0.130	−0.099	−0.110	−0.151
	***p***	^****^	^****^	^****^	^****^	^**^	^**^	NS
**KLRG1 vs. LAG-3**	***r***	−0.003	0.004	−0.022	−0.094	−0.029	−0.001	−0.085
	***p***	NS	NS	NS	^**^	NS	NS	NS
**KLRG1 vs. TIGIT**	***r***	−0.006	−0.044	−0.053	−0.145	−0.102	−0.050	0.004
	***p***	NS	^***^	^****^	^****^	^**^	NS	NS
**KLRG1 vs. GITR**	***r***	−0.056	−0.123	−0.094	−0.059	−0.057	−0.124	−0.048
	***p***	^****^	^****^	^****^	^*^	NS	^**^	NS
**KLRG1 vs. 4-1BB**	***r***	−0.039	−0.077	−0.076	−0.144	−0.042	0.031	−0.075
	***p***	^**^	^****^	^****^	^****^	NS	NS	NS
**KLRG1 vs. OX40**	***r***	−0.047	−0.080	−0.070	−0.062	−0.029	−0.046	−0.027
	***p***	^****^	^****^	^****^	^*^	NS	NS	NS

*p*-value notation ^****^ = <0.0001, ^***^ = <0.001, ^**^ = <0.01, ^*^ = <0.05.

## DISCUSSION

Much effort in the field of immuno-oncology has involved the study of combination therapies, including combinations involving blockade of more than one T cell inhibitory receptor. Combination therapies may produce clinical benefit through the additive independent action of each therapy or may produce supra-additive efficacy (in excess of that predicted by combined independent action) [18, 21, 22], which has been called clinical synergy [18]. Recent studies have demonstrated that the vast majority of combination studies with anti-PD-1 therapies have produced no more, and sometimes less, than the benefit expected from additive independent action [18, 23, 24].

Thus, supra-additive efficacy remains a desired goal. Although it can result from the well-established concept of pharmacological synergy, other mechanisms can also produce supra-additive efficacy [19]. For example, collateral sensitivity (resistance to one drug confers susceptibility to another) can also produce clinical synergy, with antibiotic combinations used to overcome drug resistance a classic example. More generally, responses to combinations of monotherapies *a* and *b* are governed by


$$Pab=Pa+Pb-PaPb-\rho \sqrt{Pa(1-Pa)Pb(1-Pb)},$$

where *Pab* is the probability of response to the combination *ab* and *ρ* is the correlation coefficient of responses to *a* and *b* [19, 22]. Positively correlated therapies (*ρ* > 0) can produce less than additive benefit and negatively correlated (anti-correlated) therapies (*ρ* < 0) can produce supra-additive benefit.

Here, we point out that KLRG1 expression is concentrated on the most differentiated and potent CD8 T cells, unlike PD-1, and its expression is anti-correlated to PD-1 as well as all major coinhibitory and costimulatory checkpoint receptors across tumor infiltrating (TILS) CD8 T cells in a wide variety of cancers. Whereas much of the T cell inhibitory drug development efforts over the last decade have been focused on combinations of expression-correlated inhibitory receptor targets, the targeting of anti-correlated inhibitory receptors has greater potential to produce supra-additive benefit, and KLRG1 has this distinct property.

## MATERIALS AND METHODS

Public domain RNAseq gene expression data of bulk immune cell populations in normal blood (GSE107011) [25] was analyzed for pairwise Pearson correlation of PD-1 (PDCD1) with other T cell inhibitory receptors across the differentiated T cell subsets CD4 TEMRA, CD8 TEM (T effector memory) and TEMRA (effector), and γδ T cells, and in lung cancer tumor infiltrating lymphocytes (TILS) from dataset GSE99531. Correlation analysis was performed of single cell RNAseq gene expression from cancer tissue datasets used in a previously published analysis [10] including GSE72046 (melanoma) [26], GSE98638 (HCC) [27], GSE103322 (HNSCC) [28], and GSE89567 (astrocytoma) [29] and newly analyzed comprehensive datasets GSE102575 (melanoma) [30], GSE108989 (colorectal cancer) [31], and GSE99254 (non-small cell lung cancer) [32]. CD8 T cells were annotated as those T cells with non-zero expression of CD8A. Published graphical data was abstracted using https://apps.automeris.io/wpd/.
