# Correction: Anti-correlation of KLRG1 and PD-1 expression in human tumor CD8 T cells

**DOI:** 10.18632/oncotarget.28846

**Published:** 2026-02-20

**Authors:** Steven A. Greenberg

**Affiliations:** ^1^Department of Neurology, Brigham and Women’s Hospital, Harvard Medical School, Boston, MA 02115, USA

**This article has been corrected:** It was found that Figure 2, “Pearson correlation of CD8 T cell co-inhibitory receptor gene expression with PD-1 across CD8 T cells from tumor samples,” contained two identical graphs for CD8 T Cells/LAG3 and CD8 T Cells/TIGIT. The authors explained that this duplication occurred because the Prism graph for TIGIT was incorrectly linked to the LAG3 data column in the source file (GSE108989). The authors have provided the original data to the Integrity Office and corrected the Figure 2 Colorectal CD8 T Cells/TIGIT graph.

Additionally, a typo was identified in the Materials and Methods section. The accession number GSE102575 was listed incorrectly and should be GSE120575 for the Melanoma data set.

These corrections do not change the conclusions of the article. The corrected Figure 2 and the updated Materials and Methods section are provided below.

Figure 2.

## MATERIALS AND METHODS

Public domain RNAseq gene expression data of bulk immune cell populations in normal blood (GSE107011) [25] was analyzed for pairwise Pearson correlation of PD-1 (PDCD1) with other T cell inhibitory receptors across the differentiated T cell subsets CD4 TEMRA, CD8 TEM (T effector memory) and TEMRA (effector), and γδ T cells, and in lung cancer tumor infiltrating lymphocytes (TILS) from dataset GSE99531. Correlation analysis was performed of single cell RNAseq gene expression from cancer tissue datasets used in a previously published analysis [10] including GSE72046 (melanoma) [26], GSE98638 (HCC) [27], GSE103322 (HNSCC) [28], and GSE89567 (astrocytoma) [29] and newly analyzed comprehensive datasets GSE120575 (melanoma) [30], GSE108989 (colorectal cancer) [31], and GSE99254 (non-small cell lung cancer) [32]. CD8 T cells were annotated as those T cells with non-zero expression of CD8A. Published graphical data was abstracted using https://apps.automeris.io/wpd/.

Original article: Oncotarget. 2025; 16:1–8. 1-8. https://doi.org/10.18632/oncotarget.28679

**Figure 2:**
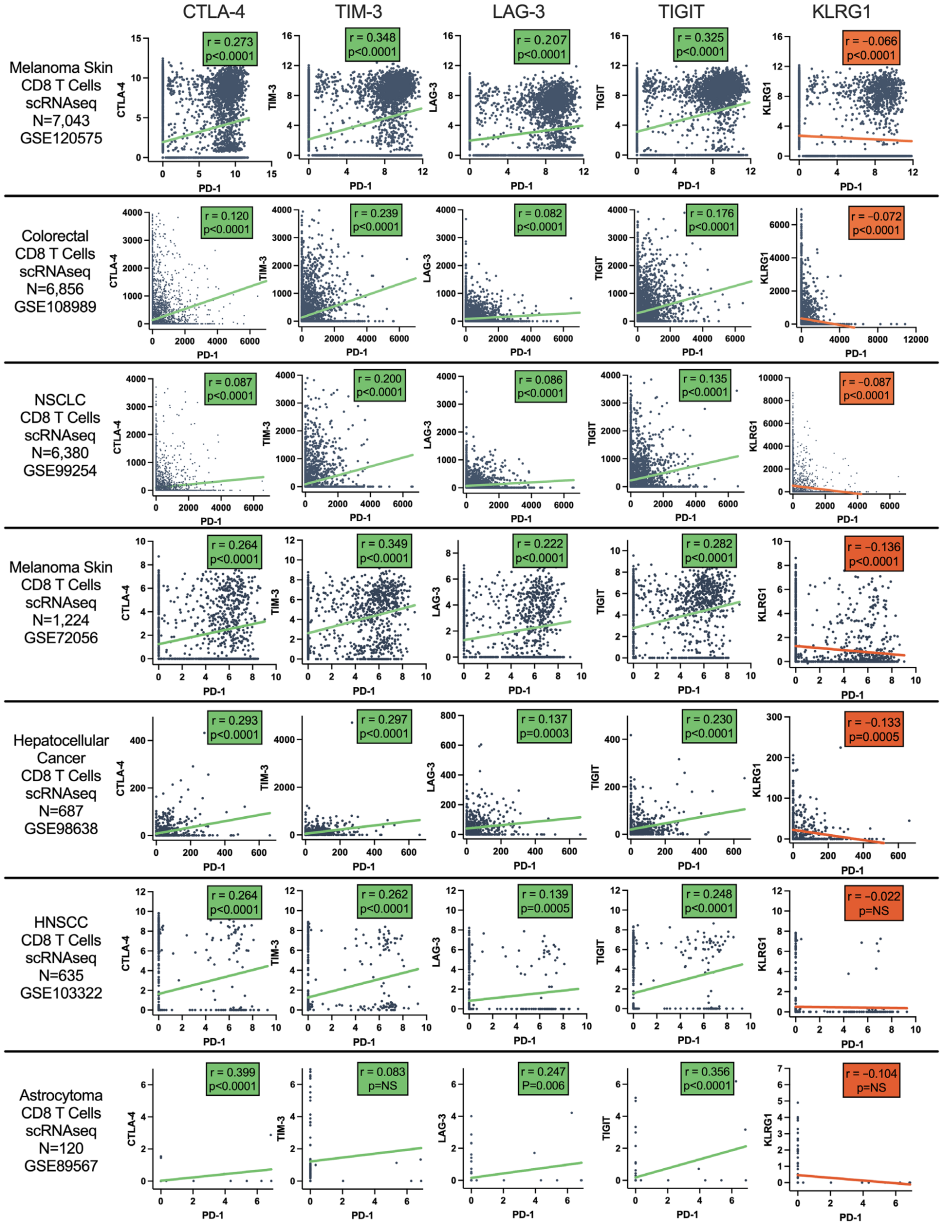
Pearson correlation of CD8 T cell co-inhibitory receptor gene expression with PD-1 across CD8 T cells from tumor samples. Data from single cell RNAseq datasets of melanoma, colorectal cancer, NSCLC, hepatocellular carcinoma (HCC), head and neck squamous cell cancer (HNSCC), and astrocytoma.

